# Patient anxiety and concern as predictors for the perceived quality of treatment and patient reported outcome (PRO) in orthopaedic surgery

**DOI:** 10.1186/1472-6963-12-244

**Published:** 2012-08-08

**Authors:** Randi Bilberg, Birgitte Nørgaard, Søren Overgaard, Kirsten Kaya Roessler

**Affiliations:** 1 Department of Orthopaedic Surgery, Kolding Hospital, a part of Lillebaelt Hospital, Skovvangen 2-6, Kolding 6000, Denmark; 2 Institute of Psychology, University of Southern Denmark, Odense, Denmark; 3 Emergency Department, Kolding Hospital, a part of Lillebaelt Hospital, Kolding, Denmark; 4 Department of Orthopaedics and Traumatology, Odense University Hospital, Odense, Denmark; 5 Institute of Clinical Research, University of Southern Denmark, Odense, Denmark

## Abstract

**Background:**

Previous studies have shown that patients’ anxiety and dissatisfaction are predictors for increased postoperative pain and reduced efficacy of pain treatment. However, it remains to be shown whether patient anxiety and concern are predictors for the perceived quality of treatment and patient reported outcome (PRO).

The aim of this study is to investigate whether there is a correlation between preoperative anxiety and concern, and the perceived quality of postoperative treatment and outcome. The hypothesis is that anxious and concerned patients are less satisfied with treatment and have a poorer outcome.

**Methods/design:**

This study is designed as a prospective follow-up study and has the aim of investigating the correlation between patient anxiety and concern, patients´ perceived quality of treatment and outcome. This correlation will be detected using five questionnaires: CMD-SQ (Common Mental Disorders Screening Questionnaire), EuroQol 5 Dimensions (EQ-5D), Short form 12 (SF-12), “What is your evaluation of the patient progress in the Department of Orthopaedic Surgery?” (HVOK), Questionnaire for patients who have had hip surgery (RCS) and Oxford Hip Score (OHS) or Oxford Shoulder Score (OSS). The patients will complete the above mentioned questionnaires preoperatively in the outpatient department, and postoperatively just before discharge from the inpatient department, and 12 and 52 weeks after the operation. The study includes a reliability test of CMD-SQ regarding this specific population and tested by means of a Kappa. A total of 500 hip- and shoulder-patients will be included from October 2010 till October 2011.

**Discussion:**

If a correlation between patient anxiety and concern, patients´ perceived quality of treatment and patient reported outcome is found, it will be recommended to screen all hip- and shoulder-patients for anxiety and concern preoperatively. Besides, it would be relevant to carry out investigations of possible interventions towards anxious and concerned patients.

**Trial registration:**

Current Controlled Trials: NCT01205295

## Background

Studies of patients´ reported quality of treatment has shown in general, that patients are very satisfied with their treatment [1-3]. However, studies investigating the correlation between patients´ satisfaction with treatment and their perceived outcome are warranted.

The overall aim of orthopaedic treatment is to reduce pain, improve functional ability and thereby enhance quality of life [4]. A study based on the Oxford Hip Score (OHS) showed a significant correlation between the clinical outcome measured by OHS, patients´ perceived quality of life (EQ-5D), and their satisfaction with treatment [5] following insertion of a total hip arthroplasty (THA). In addition, a Swedish study showed that preoperative depression and anxiety (detected by EQ-5D) had a significant correlation with postoperative satisfaction and pain as regards THA patients [6]. The patients, who were most dissatisfied with the treatment and suffered from anxiety or depression, had the smallest pain relief. However, the study underscored the need for future research by means of a more specific and sensitive questionnaire for detecting anxiety and depression preoperatively, as the study was a prospective registry trial, without opportunity for further data collection. Thus, it would be relevant to conduct a study using a more sensitive questionnaire to detect anxiety, depression and concern and their influence on postoperative patient reported outcome (PRO).

Another studies have shown a correlation between dissatisfaction with therapy, and moderate to severe postoperative pain [7,8]. Studies have shown a significant correlation between anxiety, mood and chronic pain [9,10], and that patients are 2–3 times more likely to develop chronic pain if they already suffer from a kind of depression [11,12].

A Danish study conducted by general practitioners showed a significant correlation between patients' somatoform disorder and their satisfaction with treatment [13]. Another study based on the Common Mental Disorders (CMD-SQ) questionnaire showed that it is possible to identify anxious and concerned patients and then focus on their treatment [14].

Some studies have revealed that far from all patients get the relief they are searching for by traditional therapies such as surgery and medical treatment [15,16], and that there are patients who do not receive the warranted treatment in health services [17,18]. If it is possible to identify these patients, a more targeted treatment could be used in a combination with the traditional treatment.

However, it remains to be shown whether patient anxiety and concern are predictor factors for the perceived quality of treatment and the patient reported outcome. This project is expected to reveal more precise information on the correlation between patient anxiety and concern and patients reported outcome (PRO) than previous studies have shown.

### Aim and hypothesis

The aim of this study is to investigate whether there is a correlation between patient anxiety and concern, and patients´ perceived quality and outcome of treatment. The hypothesis is that patients who are anxious and concerned are less satisfied with their treatment and have a poorer outcome.

### Material

#### Conceptual clarifications

Anxiety is an unpleasant state of mind which reduces the individuals’ ability to function in daily life. Anxiety can vary in different modes of expression, such as: agoraphobia, social phobia, simple phobia, panic disorder, generalized anxiety, obsessive-compulsive disorder, acute stress disorder and post-traumatic stress disorder [19].

Concern is defined as thoughts about things or events that may occur in life which can be positive or negative. These thoughts cause restlessness and a fear of harming one's own health and may reduce patients´ control and disable them [20]. In this study anxiety and concern are detected by the Common Mental Disorders - Screening Questionnaire (CMD-SQ) and EQ-5D.

CMD-SQ (Common Mental Disorders – Screening Questionnaire) contains 38 items and was prepared as a tool for general practitioners to increase the focus on patients` anxiety and concern. The questionnaire was translated into Danish and then validated without the test-retest. The validation tests included a total of 701 patients and the results of the CMD-SQ were compared to the SCAN-interview which was used as a golden standard. The analysis showed a Kappa of 0.86 [14,21,22]. CMD-SQ consists of the following six subscales, each of them has been validated: SCL-SOM, Whiteley-7, SCL-ANX4, SCL-8, SCL-DEF6 and CAGE [14]. The patients respond on at five point Likert scale. A normal sum score is lower than four in the SCL-AS scale and no more than two in the remaining scales based on the cut points on the ROC curve. A manual for assessing and validating the score of CMD-SQ is available [14].

EQ-5D (EuroQol 5 Dimensions) is designed to assess the health related quality of life with no reference to a specific diagnosis. The questionnaire has been translated into Danish and validated [23]. The scale includes five broad areas and a visual analogue scale. The self-reported health situation is reported on a scale from 0 to 100. The score 100 corresponds to the best self-reported health situation [24].

The perceived quality is the patients´ satisfaction with treatment detected by the questionnaire HVOK, including patients admitted to the Department of Orthopaedic Surgery. HVOK is the Danish acronym for the title which translated into English is “What is your evaluation of the patient progress in the Department of Orthopaedic Surgery?”

The Danish questionnaire HVOK includes 46 items and deals with patients' priorities and satisfaction with treatment and is included in a revised form. The questionnaire has been pilot tested at 38 patients [25]. Ten of the highest prioritised items were selected for this study. The patients respond on at five point Likert scale.

The Royal College of Surgeons of England has developed the "Questionnaire for patients who have had hip surgery" (RCS). The aim was to investigate patients´ satisfaction with surgery [26]. It is relevant to include three items as supplementary questions in this study. The patients respond on at a five point Likert scale. This three items are 1) “In general, would you say your health is?”, 2) “How will you describe the result of your operation?” and 3) “Overall, how are the problems now in the hip on which you had surgery, compared to before your operation?”

Patient reported outcome (PRO) is defined as the patients outcome of the treatment measured by SF-12 (Short Form 12) and OHS (Oxford Hip Score) or OSS (Oxford Shoulder Score) [27].

The UK questionnaires OHS and OSS are similar, but related to two different categories of patients, namely THA (Total Hip Arthoplasty) and shoulder-operated patients. The questionnaires are compiled and validated in Oxford University Hospital. Each questionnaire contains 12 items and both scores have been translated into Danish [28]. The OSS has been translated into Danish and validated [29]. The patients respond on at five point Likert scale and the sum scores can be between 12 and 60 points. 12 points reflects the best possible outcome, and a score above 36 is categorized as an expression of a poor patient-assessed outcome [27].

SF-12 is an internationally and nationally validated questionnaire that evaluates patients' self-reported health perception. SF-12 is an abbreviated version of SF-36 and it contains 12 items, which are divided into physical and mental items. The patients respond on a Likert scale [30-32].

### Charlson Co-morbidity Index

*A medical professional assessment of patients' expected co-morbidities* Charlson Co-morbidity Index is a validated method for calculating co-morbidity for each patient [33,34]. The information contained in the co-morbidity calculation is retrieved from the National Patient Registry. The Charlson Co-morbidity Index is divided into three parts where the first part is patients who are not registered in the National Patient Registry and have no co-morbidity (point 0); the second part is patients who are registered with mild to moderate co-morbidity (1–2 point) and the third part is patients who are those with severe co-morbidity [35]. It would be atypical if the study population is a heterogeneous group of patients, but the co-morbidity can be a way of making the study population more heterogeneous. The Charlson Co-morbidity Index has in other Danish and international studies proved to be an important confounder that should be adjusted for, or used as a predictor [36-38].

#### Population

Patients scheduled for a total hip arthroplasty (THA).

This group represents a part of the adult population suffering from chronic pain. It has been shown that five to ten percent of these patients suffer from severe chronic pain postoperatively [39].

Patients scheduled for a shoulder-operation.

This group was chosen because it constitutes a group of younger patients suffering from pain. Previously, they have been identified as a group who tend to develop anxiety and concern [17].

#### Inclusion criteria

All hip- and shoulder-patients who are referred for the first time to the Department of Orthopaedic Surgery at Kolding Hospital, a part of Lillebaelt Hospital and the Department of Orthopaedics and Traumatology, Odense University Hospital, Denmark are included. The patients must be able to speak and read Danish and must be at least 18 years old. To be included, they must enter a patient programme that implies an operation.

#### Exclusion criteria

Patients with primary and secondary bone tumour or who are registered as terminal are excluded. Patients who have experienced a trauma against the shoulder within the past four weeks and those diagnosed with severe mental disorders such as schizophrenia, paranoid psychosis and bipolar affective disorders are excluded.

#### Sample size

A previous study showed a significant improvement in EQ-5D after both hip operations, THA’s and shoulder operations and based on these results, this study will include a total of 202 shoulder patients and 36 THA patients [40]. This corroborates the sample size and power estimation of another study assessing OHS, SF-12 and EQ-5D at baseline and 12 months after an operation [41]. There are no studies showing the results of CMD-SQ before and after an operation.

It will be possible to include 250 THA patients and 250 shoulder patients in a one-year inclusion period.

## Methods

### Design

The study is designed as a prospective follow up study. The patients will complete the questionnaires, once preoperatively in the outpatient department and three times postoperatively; before discharge and 12 and 52 weeks after the operation. The inclusion period will be one year beginning in October 2010. The study includes a reliability test of CMD-SQ regarding this specific population, and investigated by means of a Kappa.

The reliability of CMD-SQ is tested by including 40 patients who will answer the CMD-SQ two times. The aim is to investigate whether the questionnaire responses will change over time regarding this specific population (test-retest). The hip patients will be asked to fill in the CMD-SQ at first in the outpatient department, when they join the Joint Care School, and next when they arrive to the inpatient department just before their operation. The main issue is that no essential changes had happened to the patients between the two measurements.

After the test-retest of the CMD-SQ, patients will be included for the next part of the study. When the hip patients arrive to the Joint Care School, they are asked to fill in the questionnaires: CMD-SQ, EQ–5D, SF–12, RCS and OHS. The shoulder patients are asked to fill in the questionnaires: CMD-SQ, EQ-5D, SF-12 and OSS when they arrive to the outpatient department for the first time. Both groups – hip and shoulder patients will be asked to fill in the questionnaire HVOK just before their discharge from the inpatient department. The patients are asked to fill in the questionnaires: CMD-SQ, EQ-5D, SF-12, RCS, HVOK and OHS/OSS 12 and 52 weeks after the operation. Postoperatively, the questionnaires are sent to the patients by mail and the patients are asked to return the questionnaire in the enclosed stamped and addressed envelope. If the patients do not return the questionnaire in 14 days, a new questionnaire is mailed. If the second questionnaire is not returned the patients are called and asked to return the questionnaire, or asked why they do not want to fill in the questionnaire. These precautions are taken in order to increase the response rate.

### Ethics statements

The study is presented and approved of The Regional Scientific Ethical Committee for Southern Denmark and the Danish Data Protection Agency (J.nr. 2009-41-3896).

### Study variables

The analysis will be done by CMD-SQ, EQ-5D, HVOK, SF-12, RCS, OHS/OSS and the Charlson Index. Besides, social demographic data such as gender, age, weight, height, citizenship, civil status and educational level will be included.

### Statistical analysis

The test-retest reliability is analyzed by means of an Kappa and a value above 0.70 is recommended in a clinical study [42].

The data from the preoperative questionnaire, the postoperative questionnaire just before discharge from the inpatient ward, and the questionnaire data from 12 and 52 weeks after the operation, will be analysed using a multiple linear regression test in order to investigate whether there is a correlation between patients´ anxiety and concern, and their perceived quality and outcome of treatment. It is assumed that the data are normally distributed and the will be made to different Multiple Linear Regression analysis where the first will be between baseline and after 12 weeks and the second between baseline and 52 weeks.

The results of the CMD-SQ before the operation and 3 and 12 months postoperatively, are considered the primary outcome, whereas the results of SF-12, EQ-5D, OHS/OSS, RCS and HVOK are the secondary outcome. The main result is a correlation between the primary outcome and the secondary outcomes. The analysis will be done separately and without mutual influence.

Data will be tested for confounder using the Charlson Index and social demographic data (i.e.: gender, age, weight, height, citizenship, civil status and educational level).

Missing data will be labelled as missing in all the questionnaires. For the comparative analysis for the dropouts and the respondents the categorical variables as gender, citizenship, civil status, Charlson Index and educational level will be analyzed using a chi^2 test. For the continuous variables age, weight and height will be analyzed by using a t-test. Potentially dropouts after the first measurement will be analysed with respect to the social demographic data and compared to those of the patients who stay in the study. All dropout analysis will be done by means of an unpaired t-test.

All statistical analyses are done using Stata, version 11.

## Discussion

The use of preoperative evaluation of patient anxiety and concern has not been done routinely in shoulder and hip surgery. This study aims to investigate the correlation between patient anxiety and concern and postoperative outcome. The hypothesis is that anxious and concerned patients are less satisfied with their treatment and have a poorer overall outcome of their treatment. If it is possible to confirm this hypothesis, it will be recommended to screen all hip- and shoulder-patients for anxiety and concern preoperatively. This will make it possible to optimize patient information, and to select patients with the likelihood of having a good patient reported outcome postoperatively.

The study is dependent on the fact that all patients fill in the questionnaire four times, and for that reason the study can be challenged by dropouts. Therefore, in order to get 202 patients (i.e. the required sample size) it is planned to include 250 patients. Experiences from other studies using the same versions of the PRO questionnaires have shown close to a 90 percent response rate which makes our patient number sufficient as planned in the power calculation [6,43].

It is decided to send the questionnaire to the patients 12 and 52 weeks after the operation as it has been shown that these periods constitutes stable periods for patients functional ability and pain in their recovery after the operation [44-47]. It is important to measure the patients when they are in a stable period because otherwise the outcome will be biased.

We have included the test-retest reliability of the CMD-SQ as this is recommended for each screening of a new population in a new context. The outcome of a reliability test, in this case a questionnaire, is a result of the data from this specific measurement in this specific patient group and not a result of the questionnaire itself [42]. Therefore the result of the reliability test of CMD-SQ in this study can be compared to the results of other reliability tests of CMD-SQ, but it can not be used directly in a different population.

After having tested the hypothesis, the next step is to investigate the opportunity to help the patients identified as anxious and concerned preoperatively, - i.e. a high score on CMD-SQ. If this study detects a relationship between patients´ anxiety and concern, their self-reported outcome of treatment and their perceived quality of progress, it will be relevant to develop a method for helping these patients before surgery.

## Abbreviations

CMD-SQ: Common Mental Disorders – Screening Questionnaire; PRO: Patients reported outcome; EQ-5D: EuroQol 5 Dimensions; SF-12: Short form 12; HVOK: “What is your evaluation of the patient progress in the Department of Orthopaedic Surgery?”; RCS: Questionnaire for patients who have had hip surgery; OHS: Oxford Hip Score; OSS: Oxford Shoulder Score; VAS-Scale: Visual Analogue Scale; SD: Standard Deviation; ICD-10: International Statistical Classification of Diseases: version 3 (WHO); SCL-SOM: Symptom Check List: somatisation subscale; Whiteley-7: A rating scale for illness worry and conviction; SCL-ANX4: Symptom Check List: subscale for anxiety; SCL-8: Symptom Check List: subscale for mental illness; SCL-DEF6: Symptom Check List: depression subscale; CAGE: A questionnaire for alcohol dependence.

## Competing interests

The authors declare that they have no competing interests.

## Authors’ contributions

All the authors have contributed to the article, but RB is the main responsible for the article. RB, SO, BN, KR: Study conception and design. RB: Data collection. RB: Data analysis. RB: Drafting of manuscript. SO, BN, KR: Critical revisions of manuscript for important intellectual content. RB: Obtaining funding. SO, BN, KR: Supervision. Other. All authors read and approved the final manuscript.

## Pre-publication history

The pre-publication history for this paper can be accessed here:

http://www.biomedcentral.com/1472-6963/12/244/prepub
